# Oral hygiene status and vascular aging in schoolchildren and their mothers

**DOI:** 10.1265/ehpm.24-00093

**Published:** 2024-08-10

**Authors:** Shogo Nakane, Yuki Ito, Kayo Kaneko, Sayaka Kato, Kyoko Minato, Takeshi Ebara, Shinji Saitoh, Mayumi Sugiura-Ogasawara, Yasuyuki Shibuya, Michihiro Kamijima

**Affiliations:** 1 Department of Occupational and Environmental Health, Nagoya City University Graduate School of Medical Sciences, Nagoya, Japan; 2 Department of Maxillofacial Surgery, Nagoya City University Graduate School of Medical Sciences, Nagoya, Japan; 3 Department of Ergonomics, Institute of Industrial Ecological Sciences, University of Occupational and Environmental Health, Kitakyushu, Japan; 4 Department of Pediatrics and Neonatology, Nagoya City University Graduate School of Medical Sciences, Nagoya, Japan; 5 Department of Obstetrics and Gynecology, Nagoya City University Graduate School of Medical Sciences, Nagoya, Japan

**Keywords:** Blood pressure, Child, Japan Environment and Children’s Study (JECS), Mother, Oral hygiene, Vascular stiffness

## Abstract

**Background:**

Poor oral hygiene, generally manifesting as dental caries, gingivitis, or periodontitis, is a common chronic condition among both children and adults worldwide and has been reportedly associated with hypertension and arterial stiffness mainly in adult patients. However, these associations have not been well-studied in children and adults in the general population. Therefore, we conducted this cross-sectional study to clarify the associations between oral hygiene indices and high blood pressure (BP)/hypertension and arterial stiffness as assessed by the cardio-ankle vascular index (CAVI) in children along with their mothers. The association between maternal oral hygiene and high BP in children was also examined based on the hypothesis that maternal awareness of oral hygiene is related to their children’s oral hygiene.

**Methods:**

This study was conducted as an Adjunct Study of the Aichi Regional Sub-Cohort of the Japan Environment and Children’s Study. Participating children (n = 220, 85–104 months old) and their mothers (n = 217, 29–52 years old) underwent dental/intra-oral examination and BP and CAVI assessment. High BP in children and hypertension in mothers were diagnosed according to corresponding American guidelines. Logistic regression analysis or analysis of covariance was used to examine the associations of poor oral hygiene indices with BP and CAVI.

**Results:**

Maternal dental caries ≥1 was associated with their hypertension (adjusted odds ratio [aOR]: 2.72, 95% confidence interval (CI): 1.12–6.61). Maternal dental plaque ≥1/3 was associated with maternal hypertension and children’s high BP (aOR, 95% CI: 4.71, 1.33–16.73 and 5.67, 1.22–25.04, respectively). Maximum pocket depth ≥4 mm was associated with children’s high BP (aOR: 6.85, 95% CI: 1.24–38.01). No associations were observed between oral hygiene indices and CAVI in children; however, there was a significant association between dental plaque and CAVI in mothers (F = 5.62, p < 0.01).

**Conclusions:**

The small sample size, especially the case number, made it necessary to refrain from drawing unambiguous conclusion. The hypothesis that warrants further investigation based on the present study results is that poor oral hygiene is associated with high BP in children and hypertension and arterial stiffness in mothers, and maternal oral hygiene is associated with high BP in children.

**Supplementary information:**

The online version contains supplementary material available at https://doi.org/10.1265/ehpm.24-00093.

## 1. Introduction

Poor oral hygiene generally manifests as dental caries, gingivitis, and periodontitis and is caused by dysbiosis—imbalances of oral flora—due to insufficient cleaning and high bacterial loads [1]. Dental caries and periodontitis are among the most common chronic diseases in both children and adults [2, 3]—2.4 billion children and 621 million adults worldwide suffer from untreated dental caries of the primary and permanent teeth, respectively [4] and approximately half of adults also have periodontitis [5].

Several recent studies have revealed associations between dental caries and hypertension in pediatric patients [6] and between periodontitis and hypertension [7–9], arterial stiffness [10, 11], and risk of cardiovascular diseases [12] in elders, or child or adult patients. However, although atherosclerosis can start to develop in the first decade of life, few studies have investigated the association of high blood pressure (BP), an early indicator of hypertension, and arterial stiffness with various indicators of poor oral hygiene in children [13]. Furthermore, over the past decade, the incidence of hypertension in the pediatric population has been increasing [14]. While high BP in children is generally defined based on BP percentile level, BP measured in children predicts future BP and primary hypertension in childhood is associated not only with obesity, but also with other cardiovascular risk factors [15]. In Japan, 43 million people suffer from hypertension, and over 100,000 cardiovascular deaths due to hypertension occur annually [16]. Therefore, from the public health perspective, it is important to prevent high BP from childhood onwards.

In school-aged children, parents’, especially in mothers’, awareness of oral hygiene influences oral health in their children [17]. Therefore, this cross-sectional study focused on mother-child pairs (the age of the child was 7–8 years) and aimed to investigate the associations between various oral hygiene indices—specifically, halitosis, dental plaque, and dry mouth—and high BP/hypertension or arterial stiffness in mother-child pairs. The associations between maternal oral hygiene and high BP in children was also examined based on the hypothesis that the oral hygiene status of school-going children is related to their mothers’ awareness about oral hygiene.

## 2. Methods

### 2.1 Study design, setting, and population

This cross-sectional study was designed as an Adjunct study of the Sub-Cohort Study of the Japan Environment and Children’s Study (JECS) conducted at the Aichi Regional Center [18]. Participants for the JECS, an ongoing nationwide prospective birth cohort study that aims to investigate the associations between environmental factors and child health and development, were recruited between January 2011 and March 2014 as previously described [19]. Five percent of the JECS participants were included in the Sub-Cohort Study [20]; the present cross-sectional study was conducted 8 years later between June 2021 and July 2022. In total, 270 child-mother pairs from the Aichi Regional Center cohort were considered eligible to participate in this study. Participants underwent blood sample collection and physical and dental/intra-oral examinations and were required to complete a questionnaire that included questions on oral care. The selection of study participants is shown in Supplemental Fig. 1. Finally, 201 children (85–104 months old) and 201 mothers (29–52 years old) (185 pairs) for complete case analyses, and 220 children and 217 mothers for multiple imputation analysis including subjects with missing covariables were eligible.

This study was conducted in accordance with the STROBE guidelines and the principles of the Declaration of Helsinki and approved by the Institutional Review Board, Nagoya City University, Japan (No. 70-19-0001). All participants provided written informed consent before inclusion.

### 2.2 Dental/intra-oral examinations

Except for halitosis measurement, all dental/intra-oral examinations were performed by the same dentist. Third molars were excluded from the analysis because of the high frequency of extractions.

For probing pocket depth (PD), the deepest point among six sites on each tooth was evaluated. Bleeding on probing (BOP) was assessed at six points on all teeth [21] using color code probe CP2 (Hu-Friedy; Chicago, USA). In children, BOP ≥10% was considered to be gingivitis [22]. Periodontitis in adults was defined as BOP ≥30% and maximum PD ≥4 mm [23]. The decayed, missing, and filled teeth (dmf/DMF) score, which is used to evaluate dental caries in both primary and permanent teeth [24], was obtained from dental examination records. Visible dental plaque was categorized as follows: none (no visible plaque), <1/3 (visible plaque on gingival margins only), or ≥1/3 (visible plaque elsewhere) [25]. Tongue coating, which is known to be a cause of halitosis and contains many bacteria and fungi related to periodontitis and caries [26], was classified into four categories as previously reported: none, 0–1/3, 1/3–2/3, and <2/3 [27]. In most cases, halitosis and oral moisture were assessed by the same examiner using the OralChroma analyzer (Nissha FIS; Osaka, Japan), which uses gas chromatography to detect three major halitosis-associate volatile sulfide components, and the Mucas^®^ device (LIFE Co.; Saitama, Japan), respectively, according to the manufacturer’s instructions. For halitosis assessment, 1 mL of intra-oral air collected using a syringe placed in the oral cavity with the lips closed for 30 s was immediately injected into the OralChroma analyzer, and the concentrations of hydrogen sulfide, methyl mercaptan, and dimethyl sulfide were determined (cut-off values: 112, 26, and 8 ppb, respectively) [28]. Participants for which the median of three Mucas^®^ measurements was less than 28.0 were designated as having dry mouth [29].

### 2.3 BP

Systolic and diastolic BP (SBP and DBP, respectively) were measured by nurses. For children, BP was measured thrice on the right upper arm using a sphygmomanometer (Welch Allyn Inc.; NY, USA) and a stethoscope by the auscultatory method according to the guideline [30] and the average of the second and third measurements was used for analysis [31]. For mothers, BP was measured once on the right upper arm using an electric medical sphygmomanometer (A&D Company; Tokyo, Japan).

According to the guideline of the American Academy of Pediatrics, high BP in children was defined as BP ≥90th percentile with reference to two types of BP tables [30]: i) a table based on normal-weight children judged by age, sex, and height and ii) a simplified screening table. Maternal hypertension was defined as SBP ≥130 or DBP ≥80 mmHg according to the American College of Cardiology/American Heart Association guideline [32] or a medical history of hypertension.

### 2.4 CAVI

CAVI is a non-invasive measurable indicator of the stiffness of the aorta and the femoral and tibial arteries. Pulse wave velocity (PWV) is considered the gold standards for arterial stiffness assessment but is strongly affected by SBP and DBP [33]. CAVI overcomes this disadvantage of PWV [34, 35]. CAVI was measured by a trained nurse using the VaSera VS-3000 system (Fukuda Denshi Co. Ltd.; Tokyo, Japan) based on the heart-ankle PWV adjusted for BP based on stiffness parameters. Participants lay in the supine position in a noise-free, air-conditioned room, and the average CAVI values measured on the right and left sides were used for analysis.

### 2.5 Data collection for other variables

The characteristics of the study participants and variables previously reported to be associated with periodontitis and hypertension [6, 7, 36] were assessed using a self-administered questionnaire. Data regarding age, sex, and maternal educational background were obtained using the JECS Main Study questionnaire. Information regarding maternal smoking status (current smoker, former smoker, or non-smoker), child passive smoking status (passive smoker or not), maternal drinking habits (≥1 time per month or not), exercise habits (≥30 min once a week or not), and medical history was obtained using the questionnaire completed by mothers on the day of the dental examination. The estimated daily salt intake was calculated based on urinalysis of sodium and creatinine (first morning void) on the day of the dental examination and anthropometric measurements.

### 2.6 Statistical analysis

Continuous and categorical variables are presented as the mean ± standard deviation values and numbers (%), respectively. Student’s *t*-test, Mann-Whitney U test, and chi-squared test or the Fisher’s exact test were used to compare participant characteristics.

Univariable and multivariable logistic regression was used to assess the association between oral hygiene and BP by estimating odds ratios (ORs) and their 95% confidence intervals (CIs). For children, a propensity score calculated using age in months, body weight, passive smoking status, and maternal educational background in the logistic regression model was used as a covariate in multivariable logistic regression [37] by considering a directed acyclic graph (Supplemental Fig. 3). For mothers, a propensity score calculated using age, body mass index (BMI), and estimated daily salt intake in the logistic regression model was used as a covariate in further analyses. For halitosis, each of the three substances was used as exposure variables.

A “maternal oral hygiene” propensity score was calculated using all indices that represent the oral hygiene status of mothers, i.e., maternal DMF score, BOP, maximum PD; hydrogen sulfide, methyl mercaptan, and dimethyl sulfide levels; dental plaque, tongue coating, and oral moisture (unitless quantitative value). Logistic regression analysis for children was performed using the “maternal oral hygiene” score as a covariable. Additionally, considering the possible relationship of maternal awareness with oral hygiene status with the oral hygiene status of their children and high BP in the children, we conducted a logistic regression analysis with maternal oral hygiene status as the explanatory variable and child’s high BP as the independent variable.

To evaluate the association between oral hygiene and arterial stiffness, we used Student’s *t*-test, chi-squared test or Fisher’s exact test, Mann-Whitney U test, Kruskal-Wallis test or one-way analysis of variance according to the oral hygiene categories. Analysis of covariance and Bonferroni post-hoc test were used to examine the differences in CAVI among oral hygiene categories, adjusted for the following propensity scores of covariables: month, age, sex, BMI, and estimated daily salt intake for children and age, BMI, estimated daily salt intake, drinking habit, and smoking status for mothers.

We performed multiple imputations for the missing values of covariates by using chained equations to obtain 20 imputed datasets and then got pooled data [38]. IBM SPSS Statistics ver. 26 was used for all statistical analyses. *p* < 0.05 was considered to be statistically significant.

## 3. Results

### 3.1 Characteristics of participants

Of the 201 children, 189 (94.0%) were in the normal BP group (SBP, DBP: 95.2 ± 7.0 and 47.1 ± 7.1 mmHg, respectively) and 12 (6.0%) were in the high BP group (SBP, DBP: 112.8 ± 4.1 and 49.9 ± 8.3 mmHg, respectively) classified based on the 90th percentile of BP in the American Academy of Pediatrics guideline values defined by age, sex, and height (Table 1 and Supplemental Table 1). Children in the high BP group had significantly higher d/D value and maximum PD; other parameters were not significantly different between the two groups. Of the 201 mothers, 169 (84.1%) were in the normal BP group (SBP, DBP: 102.6 ± 9.3 and 66.0 ± 7.2 mmHg, respectively) and 32 (15.9%) were in the hypertension group (SBP and DBP: 133.6 ± 21.1 mmHg and 86.8 ± 9.1 mmHg, respectively). Mothers in the hypertension group had significantly higher body weight, BMI, maximum PD, CAVI, and periodontitis, dental plaque, and dry mouth prevalence. Other parameters were comparable between the two groups. When classified based on the simplified screening table, 176 children (87.6%) were in the normal BP group (SBP, DBP: 94.4 ± 6.4 and 46.6 ± 7.0 mmHg, respectively) and 25 (12.4%) were in the high BP group (SBP, DBP: 109.9 ± 3.9 and 51.5 ± 7.1 mmHg, respectively), which had a higher maximum PD and ratio of groups above the cut-off value of hydrogen sulfide (Supplemental Table 2).

**Table 1:** Characteristics of children and mothers in the normal and high blood pressure/hypertension groups

A) Children					

**Variable**	**Total** **(n = 201)**		**Normal blood pressure** **(n = 189)**	**High blood pressure^a^** **(n = 12)**	***p***
Age (months)	94.2 ± 4.2	(85–104)	94.2 ± 4.2	94.7 ± 3.3	0.62
Sex					
Boys	95 (47.3)		89 (47.1)	6 (50.0)	0.84
Height (cm)	125.2 ± 5.3	(112.7–142.5)	125.1 ± 5.2	126.0 ± 6.8	0.69
Body weight (kg)	25.0 ± 4.3	(18.1–45)	24.8 ± 3.9	27.5 ± 8.6	0.31
BMI (kg/m^2^)	15.9 ± 2.0	(12.7–24.9)	15.8 ± 1.8	17.1 ± 3.7	0.26
dmf/DMF	2.0 ± 2.8	(0–13)	1.9 ± 2.8	3.3 ± 3.7	0.16
d/D = 0	142 (70.6)		137 (72.5)	5 (41.7)	0.02
d/D ≥ 1	59 (29.4)		52 (27.5)	7 (58.3)	
BOP (%)	25.8 ± 23.7	(0–95.8)	24.9 ± 22.9	39.0 ± 31.9	0.11
Max PD (mm)	2.7 ± 0.7	(2–6)	2.6 ± 0.6	3.5 ± 1.4	0.03
Dental plaque					
No plaque	79 (39.3)		77 (40.7)	2 (16.7)	0.22
0<, <1/3	93 (46.3)		86 (45.5)	7 (58.3)	
1/3≤	29 (14.4)		26 (13.8)	3 (25.0)	
Hydrogen sulfide (ppb)					
<112	59 (29.4)		57 (30.2)	2 (16.7)	0.32
112≤	142 (70.6)		132 (69.8)	10 (83.3)	
Methyl mercaptan (ppb)					
<26	74 (36.8)		70 (37.0)	4 (33.3)	0.80
26≤	127 (63.2)		119 (63.0)	8 (66.7)	
Dimethyl sulfide (ppb)					
<8	48 (23.9)		44 (23.3)	4 (33.3)	0.43
8≤	153 (76.1)		145 (76.7)	8 (66.7)	
Tongue coating					
No coating	57 (28.4)		52 (27.5)	5 (41.7)	0.56
0<, <1/3	75 (37.3)		71 (37.6)	4 (33.3)	
1/3≤	69 (34.3)		66 (34.9)	3 (25.0)	
Dry mouth^b^					
No	77 (38.3)		73 (38.6)	4 (33.3)	0.71
Yes	124 (61.7)		116 (61.4)	8 (66.7)	

B) Mothers					

**Variable**	**Total** **(n = 201)**		**Normal blood pressure** **(n = 169)**	**Hypertension** **(n = 32)^c^**	***p***
Age (years)	40.3 ± 4.5	(29–52)	40.0 ± 4.3	41.9 ± 5.0	0.05
BMI (kg/m^2^)	22.1 ± 3.7	(15.8–38.4)	21.6 ± 3.0	24.6 ± 5.5	0.01
dmf/DMF	11.1 ± 5.5	(0–26)	11.0 ± 5.5	12.2 ± 5.6	0.24
d/D = 0	157 (78.1)		136 (80.5)	21 (65.6)	0.06
d/D ≥ 1	44 (21.9)		33 (19.5)	11 (34.4)	
BOP (%)	29.7 ± 25.2	(0–100)	28.2 ± 24.5	37.9 ± 28.0	0.06
Max PD (mm)	3.9 ± 1.1	(2–10)	3.8 ± 1.0	4.5 ± 1.3	0.01
Periodontitis					
No	124 (61.7)		110 (65.1)	14 (43.8)	0.02
Yes	77 (38.3)		59 (34.9)	18 (56.3)	
Dental plaque					
No plaque	100 (49.8)		87 (51.5)	13 (40.6)	0.01
0<, <1/3	88 (43.8)		75 (44.4)	13 (40.6)	
1/3≤	13 (6.5)		7 (4.1)	6 (18.8)	
Hydrogen sulfide (ppb)					
<112	109 (54.2)		91 (53.8)	18 (56.3)	0.80
112≤	92 (45.8)		78 (46.2)	14 (43.8)	
Methyl mercaptan (ppb)					
<26	121 (60.2)		100 (59.2)	21 (65.6)	0.49
26≤	80 (39.8)		69 (40.8)	11 (34.4)	
Dimethyl sulfide (ppb)					
<8	58 (28.9)		46 (27.2)	12 (37.5)	0.24
8≤	143 (71.1)		123 (72.8)	20 (62.5)	
Tongue coating					
No coating	54 (26.9)		49 (29.0)	5 (15.6)	0.48
0<, <1/3	73 (36.3)		60 (35.5)	13 (40.6)	
1/3≤			NA^d^	NA^d^	
1/3≤, <2/3	52 (25.9)		42 (24.9)	10 (31.3)	
2/3≤	22 (10.9)		18 (10.7)	4 (12.5)	
Dry mouth^b^					
No	68 (33.8)		62 (36.7)	6 (18.8)	0.05
Yes	133 (66.2)		107 (63.3)	26 (81.3)	

Continuous variables are presented as the mean ± SD (Min–Max) and categorical variables as number (percentage).

^a^Based on the table stratified by age, sex, and height in the American Academy of Pediatrics (2017) guideline

^b^Less than the cut-off value of 28.0 or not

^c^Categorized according to the ACC/AHA guideline (2017)

^d^not applicable owing to different categorization

Most oral indices were associated with each other (e.g., maximum PD and periodontitis prevalence with dental caries and plaque deposition) (Supplemental Table 3). SBP was associated with all reduced dental scores, although average CAVI did not differ significantly among the groups, except for dental plaque deposition groups in mothers.

### 3.2 Association between oral hygiene and high BP/hypertension

In children, d/D ≥1 and maximum PD ≥4 mm were significantly associated with high BP in univariate model 1 (Table 2). The adjusted OR (aOR) of maximum PD ≥4 mm for high BP was 6.85 (95% CI: 1.24–38.01) compared with maximum PD = 2 (no participants had maximum PD = 1) after adjusting for propensity score calculated based on age, body weight, passive smoking status, and maternal educational background (model 2). These elevated ORs were constant irrespective of multiple imputations (aOR: 6.55, 95% CI: 1.24–34.67). When maternal oral hygiene status was added as a covariate in addition to the aforementioned covariates, PD ≥4 mm was associated with high BP (aOR: 6.32, 95% CI: 1.10–36.46). Maternal periodontitis and plaque ≥1/3 were significantly associated with hypertension without the adjustments. In both complete and multiple imputation analysis, D ≥1 (aOR, 95% CI: 2.72, 1.12–6.61 and 2.87, 1.21–6.82, respectively) and plaque ≥1/3 (aOR, 95% CI: 4.71, 1.33–16.73 and 4.55, 1.32–15.73, respectively) were associated with hypertension after adjustment for covariates.

**Table 2:** Odds ratios of oral hygiene indices for high blood pressure/hypertension in children and their mothers

A. Children														

**Variable**		**Complete case (n = 201)**					**Multiplicatively imputed dataset (n = 220)**					**Complete pairs (n = 185)^a^**		
		
		**High ** **BP**	**Model 1** **(univariable)**		**Model 2^b^** **(multivariable)**		**High ** **BP**	**Model 1** **(univariable)**		**Model 2^b^** **(multivariable)**		**High ** **BP**	**Model 3^c^** **(multivariable)**	
		**(n)**	**OR**	**95% CI**	**aOR**	**95% CI**	**(n)**	**OR**	**95% CI**	**aOR**	**95% CI**	**(n)**	**aOR**	**95% CI**
d/D ≥1		7	3.69	1.12–12.14	2.91	0.84–10.07	7	3.45	1.06–11.31	2.75	0.80–9.48	7	2.17	0.57–8.21
BOP ≥10%		9	1.65	0.43–6.29	1.29	0.32–5.17	9	1.62	0.43–6.18	1.42	0.37–5.60	9	1.20	0.29–4.92
Max PD^d^	3 mm	5	1.46	0.34–6.28	1.22	0.27–5.45	5	1.47	0.34–6.33	1.23	0.28–5.48	5	1.05	0.22–4.98
	4 mm≤	4	10.06	1.98–51.03	6.85	1.24–38.01	4	8.67	1.75–42.89	6.55	1.24–34.67	4	6.32	1.10–36.46
Dental plaque ≥1/3		3	2.09	0.53–8.23	2.19	0.54–8.85	3	1.98	0.51–7.73	2.24	0.56–9.02	3	1.90	0.41–8.71
Tongue coating^e^		7	0.53	0.16–1.75	0.66	0.19–2.32	7	0.52	0.16–1.69	0.64	0.18–2.26	7	0.51	0.14–1.93
Dry mouth^f^		8	1.26	0.37–4.33	1.23	0.35–4.35	8	1.18	0.34–4.03	1.15	0.33–4.06	8	1.12	0.30–4.12

B. Mothers														

**Variables**	**Complete case (n = 201)**					**Multiplicatively imputed dataset (n = 217)**				
	
	**Hypertension**	**Model 1** **(univariable)**		**Model 2^g^** **(multivariable)**		**Hypertension**	**Model 1** **(univariable)**		**Model 2^g^** **(multivariable)**	
	**(n)**	**OR**	**95% CI**	**aOR**	**95% CI**	**(n)**	**OR**	**95% CI**	**aOR**	**95% CI**
D ≥1	11	2.16	0.95–4.92	2.72	1.12–6.61	13	2.24	1.03–4.85	2.87	1.21–6.82
Periodontitis	18	2.40	1.11–5.16	1.86	0.81–4.28	21	2.90	1.38–6.10	1.92	0.85–4.35
Dental plaque ≥1/3	6	5.34	1.66–17.15	4.71	1.33–16.73	7	5.44	1.83–16.17	4.55	1.32–15.73
Tongue coating^e^	27	2.20	0.80–6.06	1.96	0.68–5.66	30	2.47	0.91–6.70	2.18	0.74–6.42
Dry mouth^f^	26	1.83	0.70–4.79	1.72	0.65–4.56	28	2.22	0.92–5.37	2.14	0.80–5.75

^a^Complete child-mother pairs

^b^Adjusted for propensity score calculated based on age (months), body weight, passive smoking status, and maternal educational background

^c^Adjusted for propensity score calculated based on age (months), body weight, passive smoking status, maternal educational background, and propensity score of maternal oral hygiene

^d^PD = 2 mm was used as a reference (no participant had max PD = 1)

^e^No coating was used as the reference

^f^Less than the cut-off value of 28.0 or not

^g^Adjusted for propensity score calculated using age and BMI, and estimated daily salt intake

The ORs for high BP in children as determined using the simplified screening table are summarized in Supplemental Table 4. Maximum PD ≥4 mm was associated with high BP in the crude model (model 1; OR, 95% CI: 7.52, 2.07–27.34) and adjusted complete case and multiple imputations (model 2; aOR, 95% CI: 5.18, 1.33–20.23 and 4.94, 1.35–18.09). After “maternal oral hygiene” was added as a covariate in model 2, the association between PD ≥4 mm and high BP disappeared.

### 3.3 Association between maternal oral hygiene indices and high BP in children

Table 3 and Supplemental Table 5 show the ORs for high BP in children when maternal oral hygiene was considered an exposure. Maternal dental plaque ≥1/3 was associated with high BP in children compared with the <1/3 group when BP was categorized based on both the table stratified by age, sex, and height (aOR: 5.67, 95% CI: 1.22–25.04; Table 3) and the simplified table for screening (aOR: 5.25, 95% CI: 1.48–18.61; Supplemental Table 5).

**Table 3:** Odds ratios of maternal oral hygiene parameters associated with high blood pressure in children

**Variables**	**Complete pairs (n = 185)**				

	**High BP**	**Model 1 (univariable)**		**Model 2^a^ (multivariable)**	
	**(n)**	**OR**	**95% CI**	**aOR**	**95% CI**
D ≥1	2	NA^b^	—	NA^b^	—
Periodontitis	5	1.22	0.37–3.99	1.07	0.31–3.68
Dental plaque ≥1/3	3	6.07	1.40–26.39	5.67	1.22–25.04
Tongue coating	12	NA^b^	—	NA^b^	—
Dry mouth^c^	9	1.51	0.40–5.80	1.45	0.38–5.60

^a^Adjusted for the propensity score calculated using maternal age, BMI, and estimated daily salt intake

^b^Not applicable as there were two or no pairs of a mother with D ≥1 or no tongue coating and a child with high blood pressure, respectively.

^c^Less than the cut-off value of 28.0 or not

### 3.4 Association between oral hygiene and CAVI in children and their mothers

There were no significant associations between oral hygiene status and CAVI in children, even after adjusting for the propensity score of the covariates (Table 4). Among mothers, the estimated marginal mean of CAVI was 6.5 ± 0.1 in the no dental plaque group (n = 100), 6.8 ± 0.1 in the dental plaque <1/3 group (n = 88), and 6.8 ± 0.2 in the dental plaque ≥1/3 group (n = 13) (F statistic = 5.62, p < 0.01). CAVI in the dental plaque <1/3 group was significantly higher than that in the group without plaque (Bonferroni analysis, p = 0.01), although this difference was not statistically significant in ≥1/3 group, probably due to the small sample size.

**Table 4:** Estimated marginal means of the CAVI according to oral hygiene indices in children and mothers

	**Variable**		**n (%)**	**Estimated marginal mean ± SE**	**F statistic**	***p***
Children	Max PD	2 mm	86 (42.8)	4.7 ± 0.1	1.76	0.17
		3 mm	100 (49.8)	4.6 ± 0.1		
		4 mm≤	15 (7.5)	4.7 ± 0.2		

	d/D	0	142 (70.6)	4.7 ± 0.1	0.61	0.44
		1≤	59 (29.4)	4.6 ± 0.1		

	Dental plaque	No plaque	79 (39.3)	4.7 ± 0.1	0.59	0.55
		0<, <1/3	93 (46.3)	4.6 ± 0.1		
		1/3≤	29 (14.4)	4.5 ± 0.1		

Mothers	Periodontitis	No	124 (61.7)	6.6 ± 0.1	2.32	0.13
		Yes	77 (38.3)	6.8 ± 0.1		

	D	0	157 (78.1)	6.7 ± 0.1	0.16	0.69
		1≤	44 (21.9)	6.7 ± 0.1		

	Dental plaque	No plaque	100 (49.8)	6.5 ± 0.1	5.62	<0.01
		0<, <1/3	88 (43.8)	6.8 ± 0.1*		
		1/3≤	13 (6.5)	6.8 ± 0.2		

ANCOVA was used to estimate marginal CAVI means among the exposure categories after adjusting for the propensity score. Children’s propensity score was calculated using age (month), sex, BMI, and estimated daily salt intake.

Maternal propensity score was calculated based on age, BMI, estimated daily salt intake, drinking habit, and smoking status. *p < 0.05 vs no plaque group

### 3.5 Association between halitosis-causing substances and high BP/hypertension

Overall halitosis was not associated with high BP in either children or mothers (Supplemental Table 6), but hydrogen sulfide level above cut-off value was associated with high BP defined using the simplified screening table (Supplemental Table 7.; aOR, 95% CI: 3.91, 1.07–14.29 in the complete case and 4.03, 1.15–14.17 in the multiplicatively imputed analysis).

## 4. Discussion

The sample size became smaller than initially planned since more participants than expected refused to participate in the present study that was conducted during the COVID-19 pandemic. The study participants, who comprised the Sub-Cohort of the JECS that included home visits at 1.5 and 3.0 years and in-person surveys every two years, tended to be more health-conscious than the general population. The oral hygiene status was thus better in general as discussed later. Under the situation, the case number was minimum, which made it necessary to refrain from drawing unambiguous conclusion. However, the following findings were considered potentially important. First, dental caries was positively associated with hypertension and dental plaque associated with hypertension and arterial stiffness in mothers. Second, PD, an indicator of gingivitis and periodontitis, was positively associated with high BP in children, whereas there was no association between periodontitis and hypertension in mothers. Third, maternal oral hygiene was associated with high BP in children.

To the best of our knowledge, this is the first study to investigate the association between poor oral hygiene and CAVI as a measure of arterial stiffness independent of BP [34, 35]. In the present study, maximum PD ≥4 mm was associated with elevated BP in children regardless of their body weight, even though no association of poor oral hygiene with CAVI was observed. BP measured in children predicts future BP and is also associated with other cardiovascular risk factor as well as obesity [15]. Additionally, childhood signs of oral infection judged based on BOP, increased PD, and caries (df/DF) are reportedly associated with carotid artery intima-media thickness in adulthood [39], suggesting that follow-up studies using CAVI are required to check for subclinical atherosclerosis later in life.

Unlike a previous study that showed a positive association between dental caries and hypertension in children [6], no such association was observed in the present study, although this could have been due to differences in the BP range and age groups between the two studies, as the previous study included participants aged 6–18 years. As for mothers, the results of the present study did not show an association between periodontitis and hypertension or arterial stiffness, which was contrary to the findings of previous studies reporting positive associations between periodontitis and hypertension or arterial stiffness in adults [11, 40, 41]. The previous studies were not conducted in the general population, which may have resulted in the discrepancy. A total of 124 (61.7%) mothers were not diagnosed with periodontitis in this study, indicating a relatively favorable periodontal tissue condition of the present study participants. No association between relatively good periodontal tissue status and arterial stiffness, and periodontitis and hypertension was found in the age ≥45 years group in the general population in the National Health 2000 Survey conducted in Finland [42], and in the U.S. National Health and Nutrition Examination Survey [43]. Thus, the relatively good periodontal tissue status in the general study subjects may have led to a null association in this study. An association between dental caries and hypertension in mothers was observed in the present study, which is in line with several studies, including a study on male Japanese workers [44].

Interestingly, the results of this study showed that maternal oral hygiene status may be partially related to high BP in children. Salt intake in children did not differ according to maternal oral hygiene status. Maternal oral hygiene status has been reported to be associated with dental caries in children [45], suggesting that it is associated with high BP in children due to dental caries. Oral hygiene has been reported to be associated with hypertension and type 2 diabetes mellitus in adults and frequent tooth brushing reduced risk in population-based cohorts in China. Schoolchildren start to take care of their teeth by themselves alone but mothers’ awareness still influenced their children’s tooth-brushing behaviors [17]. Thus, a future study is warranted to test the hypothesis that educating parents as well as their children about the importance of oral hygiene leads to prevention of various cardiovascular diseases related to oral hygiene.

This study had several limitations. First, as described in the beginning of this section, the sample size was small, thereby a wide range of CI exists, which may interfere with reproducibility. We simulated several patterns for the association of max PD 4 mm≤ with high BP in a complete case model in children (aOR: 6.85, 95% CI: 1.24–38.01). If one high BP case decreased/increased and the one normotensive control increased/decreased, respectively, under the condition that the total number remained the same, the results (aOR and 95%CI: 9.86, 1.81–53.52 and 8.70, 1.63–46.55, respectively) would be similar to the actual values. However, if one high BP case increased in max PD 2 mm and 3 mm groups for each and one normotensive control decreased in the respective groups, 95%CI would contain 1 (aOR: 4.88, 95% CI: 0.96–24.95). The lower and upper limits of 95%CI fluctuated depending on the propensity score as well. Thus, the results could be statistically insignificant although the aOR is expected to be higher than 1. Second, the e-values for the point estimates of the association with a statistical significance are quite high (4.5–20.7), but low for the lower limit of CI (1.4–2.3), indicating that certain unknown confounding factors might explain the association, while it is unclear which confounding factor would be accounted for in the analysis. Additionally, the possibility of Type I errors occurring overall cannot be ruled out due to the number of tests performed. Moreover, causal relationships could not be established given the cross-sectional study design; thus, further follow-up studies are necessary for validating these results. Finally, the participants tended to be more health-conscious than the general population. Thus, although the JECS is designed to have a low bias, the results may not have high external validity. The strengths of this study include simultaneous early arterial stiffness measurement in child-mother pairs, which enabled the investigation of the associations between maternal oral hygiene and BP in children for the first time.

## Conclusions

In summary, although the small sample size, especially the case number, made it necessary to refrain from drawing unambiguous conclusion, the hypothesis that poor oral hygiene is associated with high BP in general schoolchildren and hypertension and arterial stiffness in their mothers, and that maternal oral hygiene status is related to high BP in children, warrants further investigation. Follow-up prospective cohort studies should be conducted to validate these relationships.

## Abbreviations

ACC/AHA: American College of Cardiology/American Heart Association
ANCOVA: analysis of covariance
aOR: adjusted OR
BMI: body mass index
BOP: bleeding on probing
BP: blood pressure
CAVI: cardio-ankle vascular index
CI: confidence interval
DBP: diastolic blood pressure
dmf: decayed missing filled teeth
JECS: Japan Environment and Children’s Study
OR: odds ratio
PD: pocket depth
PWV: pulse wave velocity
SD: standard deviation
SE: standard error
SBP: systolic blood pressure

## Supplementary information

**Additional file 1:** **Supplemental Table 1.** Characteristics of outcome and covariates of children and mothers in the normal and high blood pressure groups. **Supplemental Table 2.** Characteristics of children with normal or high blood pressure as defined by a simplified table. **Supplemental Table 3.** Differences in participant characteristics among groups stratified by oral hygiene index categories. **Supplemental Table 4.** Odds ratios for high blood pressure in children defined using the simplified table. **Supplemental Table 5.** Odds ratios of maternal oral hygiene parameters for high blood pressure in children defined using the simplified table. **Supplemental Table 6.** Odds ratios of halitosis-causing substances for high blood pressure. **Supplemental Table 7.** Odds ratios of halitosis-causing substances for high blood pressure in children as defined using the simplified table. **Supplemental Table 8.** Odds ratios of oral hygiene indices for high blood pressure in children and their mothers adjusted for covariates individually. **Supplemental Table 9.** Odds ratios of maternal oral hygiene parameters associated with high blood pressure in children adjusted for covariates individually. **Supplemental Table 10.** Estimated marginal means of the CAVI according to oral hygiene indices in children and mothers adjusted for covariates individually. **Supplemental Fig. 1.** Study participant selection flowchart. **Supplemental Fig. 2.** Directed acyclic graph used for the analyses. **Supplemental Fig. 3.** Distribution of propensity scores per group in children’s oral hygiene indices. **Supplemental Fig. 4.** Distribution of propensity scores per group in mother’s oral hygiene indices.

## Declaration

### Availability of data and material

Data are unsuitable for public deposition due to ethical restrictions and legal framework of Japan. It is prohibited by the Act on the Protection of Personal Information (Act No. 57 of 30 May 2003, amendment on 9 September 2015) to publicly deposit the data containing personal information. Ethical Guidelines for Medical and Health Research Involving Human Subjects enforced by the Japan Ministry of Education, Culture, Sports, Science and Technology, and the Ministry of Health, Labour and Welfare also restrict the open sharing of the epidemiologic data. All inquiries about access to the JECS Main Study data should be sent to: jecsen@nies.go.jp. The person responsible for handling enquiries sent to this e-mail address is Dr. Shoji F. Nakayama, JECS Programme Office, National Institute for Environmental Studies.
