# Supplementary material for: Oral hygiene status and vascular aging in schoolchildren and their mothers
Source: Environ Health Prev Med. 2024 Aug 10;29:42. doi: 10.1265/ehpm.24-00093 (PMC11341943; doi:10.1265/ehpm.24-00093)
Supplement: Supplementary file 1 — Additional file 1: Supplemental Table 1. Characteristics of outcome and covariates of children and mothers in the normal and high blood pressure groups. Supplemental Table 2. Characteristics of children with normal or high blood pressure as defined by a simplified table. Supplemental Table 3. Differences in participant characteristics among groups stratified by oral hygiene index categories. Supplemental Table 4. Odds ratios for high blood pressure in children defined using the simplified table. Supplemental Table 5. Odds ratios of maternal oral hygiene parameters for high blood pressure in children defined using the simplified table. Supplemental Table 6. Odds ratios of halitosis-causing substances for high blood pressure. Supplemental Table 7. Odds ratios of halitosis-causing substances for high blood pressure in children as defined using the simplified table. Supplemental Table 8. Odds ratios of oral hygiene indices for high blood pressure in children and their mothers adjusted for covariates individually. Supplemental Table 9. Odds ratios of maternal oral hygiene parameters associated with high blood pressure in children adjusted for covariates individually. Supplemental Table 10. Estimated marginal means of the CAVI according to oral hygiene indices in children and mothers adjusted for covariates individually. Supplemental Fig. 1. Study participant selection flowchart. Supplemental Fig. 2. Directed acyclic graph used for the analyses. Supplemental Fig. 3. Distribution of propensity scores per group in children’s oral hygiene indices. Supplemental Fig. 4. Distribution of propensity scores per group in mother’s oral hygiene indices. [file ehpm-29-042-s001.docx]

**Supplementary Materials**

**Oral hygiene and vascular aging in school-aged children and their mothers**

**Shogo Nakane^1, 2^, Yuki Ito^1,^ *, Kayo Kaneko^1^, Sayaka Kato^1^, Kyoko Minato^1^, Takeshi Ebara^1, 3^, Shinji Saitoh^4^, Mayumi Sugiura-Ogasawara^5^, Yasuyuki Shibuya^2^, Michihiro Kamijima^1,^ ***

^1^Department of Occupational and Environmental Health, Nagoya City University Graduate School of Medical Sciences, Nagoya, Japan

^2^Department of Maxillofacial Surgery, Nagoya City University Graduate School of Medical Sciences, Nagoya, Japan

^3^Department of Ergonomics, Institute of Industrial Ecological Sciences, University of Occupational and Environmental Health, Kitakyushu, Japan

^4^Department of Pediatrics and Neonatology, Nagoya City University Graduate School of Medical Sciences, Nagoya, Japan

^5^Department of Obstetrics and Gynecology, Nagoya City University Graduate School of Medical Sciences, Nagoya, Japan

*Corresponding authors: Yuki Ito and Michihiro Kamijima

Department of Occupational and Environmental Health, Nagoya City University Graduate School of Medical Sciences, 1 Kawasumi, Mizuho-cho, Mizuho-ku, Nagoya, 467-8601, Japan

E-mail: yukey@med.nagoya-cu.ac.jp (Y. Ito), kamijima@med.nagoya-cu.ac.jp (M. Kamijima)

Supplemental Table 1. Characteristics of outcome and covariates of children and mothers in the normal and high blood pressure groups

A) Children

|  |  | Total  (n=201) | | Normal blood pressure (n=189) | High blood pressure ^a^ (n=12) | *p* |
| --- | --- | --- | --- | --- | --- | --- |
| Variable | |  |  |  |  |  |
| SBP (mmHg) | | 96.3±8.0 | (79–121) | 95.2±7.0 | 112.8±4.1 | <0.01 |
| DBP (mmHg) | | 47.3±7.2 | (31–66) | 47.1±7.1 | 49.9±8.3 | 0.27 |
| CAVI |  | 4.6±0.6 | (2.9–7.3) | 4.6±0.6 | 4.5±0.6 | 0.47 |
| Exercise habits^b^ | |  |  |  |  |  |
|  | No | 69 (34.3) | | 66 (34.9) | 3 (25.0) | 0.48 |
|  | Yes | 132 (65.7) | | 123 (65.1) | 9 (75.0) |  |
| Passive smoking | |  |  |  |  |  |
|  | No | 166 (82.6) | | 157 (83.1) | 9 (75.0) | 0.47 |
|  | Yes | 35 (17.4) | | 32 (16.9) | 3 (25.0) |  |
| Maternal education | |  |  |  |  |  |
|  | Junior high school/high school | 39 (19.4) | | 37 (19.6) | 2 (16.7) | 0.42 |
|  | Specialized high school/vocational school/college | 82 (40.8) | | 75 (39.7) | 7 (58.3) |  |
|  | University/graduate school | 80 (39.8) | | 77 (40.7) | 3 (25.0) |  |
| Estimated daily salt intake (g) | | 6.5±1.2 | (3.3–10.1) | 6.5±1.2 | 6.3±1.3 | 0.73 |

B) Mothers

|  |  | Total  (n=201) | |  | Normal blood pressure (n=169) | Hypertension (n=32)^c^ | *p* |
| --- | --- | --- | --- | --- | --- | --- | --- |
| Variable | |  |  |  |  |  |  |
| SBP (mmHg) | | 107.5±16.5 | (80–200) |  | 102.6±9.3 | 133.6±21.1 | <0.01 |
| DBP (mmHg) | | 69.3±10.7 | (43–106) |  | 66.0±7.2 | 86.8±9.1 | <0.01 |
| CAVI |  | 6.7±0.7 | (3.9–8.8) |  | 6.6±0.7 | 6.9±0.7 | 0.03 |
| Exercise habits^b^ | |  |  |  |  |  |  |
|  | No | 163 (81.1) | |  | 137 (81.1) | 26 (81.3) | 0.98 |
|  | Yes | 38 (18.9) | |  | 32 (18.9) | 6 (18.8) |  |
| Smoking | |  |  |  |  |  |  |
|  | Non smoker | 158 (78.6) | |  | 135 (79.9) | 23 (71.9) | 0.40 |
|  | Former smoker | 33 (16.4) | |  | 27 (16.0) | 6 (18.8) |  |
|  | Smoker | 10 (5.0) | |  | 7 (4.1) | 3 (9.4) |  |
| Passive smoking | |  |  |  |  |  |  |
|  | No | 170 (84.6) | |  | 143 (84.6) | 27 (84.4) | 0.97 |
|  | Yes | 31 (15.4) | |  | 26 (15.4) | 5 (15.6) |  |
| Drinking habits^d^ | |  |  |  |  |  |  |
|  | Non drinker | 113 (56.2) | |  | 96 (56.8) | 17 (53.1) | 0.70 |
|  | Drinker | 88 (43.8) | |  | 73 (43.2) | 15 (46.9) |  |
| Maternal education | |  |  |  |  |  |  |
|  | Junior high school/high school | 40 (19.9) | |  | 33 (19.5) | 7 (21.9) | 0.10 |
|  | Specialized high school/vocational school/college | 78 (38.8) | |  | 61 (36.1) | 17 (53.1) |  |
|  | University/graduate school | 83 (41.3) | |  | 75 (44.4) | 8 (25.0) |  |
| Estimated daily salt intake (g) | | 8.1±1.7 | (2.8–13.3) |  | 8.1±1.6 | 8.6±2.2 | 0.19 |

Continuous variables are presented as the mean±SD (Min–Max) and categorical variables as number (percentage).

^a^Based on the table stratified by age, sex, and height in the American Academy of Pediatrics (2017) guideline

^b^At least 30 min of exercise once a week or not

^c^Categorized according to the ACC/AHA guideline (2017)

^d^Non-drinkers were defined as those who drank less than once per month

Supplemental Table 2. Characteristics of children with normal or high blood pressure as defined by a simplified table

| Variable | | Normal (n=176) | High blood pressure (n=25)^a^ | *p* |
| --- | --- | --- | --- | --- |
| Age (months) | | 94.0±4.3 | 95.2±3.3 | 0.12 |
| Sex | |  |  |  |
|  | Male | 81 (46.0) | 14 (56.0) | 0.35 |
|  | Female | 95 (54.0) | 11 (44.0) |  |
| Height (cm) | | 125.0±5.3 | 126.5±5.0 | 0.15 |
| Weight (kg) | | 24.7±3.8 | 27.1±6.6 | 0.08 |
| BMI (kg/m^2^) | | 15.7±1.7 | 16.8±3.0 | 0.10 |
| dmf/DMF | | 1.8±2.6 | 3.1±3.9 | 0.13 |
|  | d/D | 0.5±1.2 | 1.0±1.8 | 0.14 |
|  | m/M | 0.0±0.5 | 0.0±0.0 | 0.71 |
|  | f/F | 1.3±2.0 | 2.1±2.6 | 0.17 |
|  | d/D =0 | 127 (72.2) | 15 (60.0) | 0.21 |
|  | d/D >0 | 49 (27.8) | 10 (40.0) |  |
| BOP (%) | | 24.2±22.4 | 36.7±29.7 | 0.06 |
| Max PD (mm) | | 2.6±0.6 | 3.2±1.2 | 0.01 |
| Hydrogen sulfide (ppb) | |  |  |  |
|  | <112 | 56 (31.8) | 3 (12.0) | 0.04 |
|  | 112≤ | 120 (68.2) | 22 (88.0) |  |
| Methyl mercaptan (ppb) | |  |  |  |
|  | <26 | 66 (37.5) | 8 (32.0) | 0.59 |
|  | 26≤ | 110 (62.5) | 17 (68.0) |  |
| Dimethyl sulfide (ppb) | |  |  |  |
|  | <8 | 42 (23.9) | 6 (24.0) | 0.99 |
|  | 8≤ | 134 (76.1) | 19 (76.0) |  |
| Dry mouth^b^ | |  |  |  |
|  | No | 68 (38.6) | 9 (36.0) | 0.80 |
|  | Yes | 108 (61.4) | 16 (64.0) |  |
| Tongue coating | |  |  |  |
|  | No coating | 47 (26.7) | 10 (40.0) | 0.32 |
|  | 0<, <1/3 | 66 (37.5) | 9 (36.0) |  |
|  | 1/3≤ | 63 (35.8) | 6 (24.0) |  |
| Dental plaque | |  |  |  |
|  | No plaque | 72 (40.9) | 7 (28.0) | 0.25 |
|  | <1/3 | 81 (46.0) | 12 (48.0) |  |
|  | 1/3≤ | 23 (13.1) | 6 (24.0) |  |
| SBP (mmHg) | | 94.4±6.4 | 109.9±3.9 | <0.01 |
| DBP (mmHg) | | 46.6±7.0 | 51.5±7.1 | <0.01 |
| CAVI |  | 4.6±0.6 | 4.6±0.5 | 0.63 |
| Exercise habits^c^ | |  |  |  |
|  | No | 61 (34.7) | 8 (32.0) | 0.79 |
|  | Yes | 115 (65.3) | 17 (68.0) |  |
| Passive smoking | |  |  |  |
|  | No | 148 (84.1) | 18 (72.0) | 0.14 |
|  | Yes | 28 (15.9) | 7 (28.0) |  |
| Maternal education | |  |  |  |
|  | Junior high school, high school | 34 (19.3) | 5 (20.0) | 0.67 |
|  | Specialized high school, vocational school, college | 70 (39.8) | 12 (48.0) |  |
|  | University, graduate school | 72 (40.9) | 8 (32.0) |  |
| Estimated daily salt intake (g) | | 6.5±1.2 | 6.4±1.2 | 0.91 |

Continuous variables, mean±SD; categorical variables, n (%)

^a^Based on 90%ile of blood pressure stratified by age, sex, and 5%ile of height according to initial screening tables by the American Academy of Pediatrics (2017)

^b^Less than the cut-off value of 28.0 or not

^c^At least 30 min of exercise once a week or not

BMI, body mass index; BOP, bleeding on probing; CAVI, cardio-ankle vascular index; DBP, diastolic blood pressure; dmf, decayed missing filled teeth; PD, pocket depth; SBP, systolic blood pressure

Supplemental Table 3. Differences in participant characteristics among groups stratified by oral hygiene index categories

A. Children

|  |  | d/D | |  |  | Max PD | | |  |  | Dental plaque | | |  |
| --- | --- | --- | --- | --- | --- | --- | --- | --- | --- | --- | --- | --- | --- | --- |
|  |  | 0 | 1≤ |  |  | 2 mm | 3 mm | 4 mm≤ |  |  | No | 0<, <1/3 | 1/3≤ |  |
| Variable | | n=142 | n=59 | *p* |  | n=86 | n=100 | n=15 | *p* |  | n=79 | n=93 | n=29 | *p* |
| Age (months) | | 93.9±4.2 | 94.9±4.0 | 0.09 |  | 93.2±4.4 | 94.7±3.8 | 96.1±3.9 | 0.01 |  | 93.9±4.4 | 93.9±3.9 | 95.8±4.1 | 0.07 |
| Sex | |  |  |  |  |  |  |  |  |  |  |  |  |  |
|  | Male | 62 (43.7) | 33 (55.9) | 0.11 |  | 32 (37.2) | 53 (53.0) | 10 (66.7) | 0.03 |  | 29 (36.7) | 48 (51.6) | 18 (62.1) | 0.03 |
|  | Female | 80 (56.3) | 26 (44.1) |  |  | 54 (62.8) | 47 (47.0) | 5 (33.3) |  |  | 50 (63.3) | 45 (48.4) | 11 (37.9) |  |
| BMI (kg/m^2^) | | 15.7±1.7 | 16.2±2.4 | 0.18 |  | 15.4±1.3 | 16.0±2.0 | 17.7±3.4 | <0.01 |  | 15.6±1.6 | 16.0±2.2 | 16.0±2.0 | 0.39 |
| dmf/DMF | |  |  |  |  |  |  |  |  |  |  |  |  |  |
|  | d/D=0 | ― | ― |  |  | 70 (81.4) | 67 (67.0) | 5 (33.3) | <0.01 |  | 69 (87.3) | 59 (63.4) | 14 (48.3) | <0.01 |
|  | d/D≥1 | ― | ― |  |  | 16 (18.6) | 33 (33.0) | 10 (66.7) |  |  | 10 (12.7) | 34 (36.6) | 15 (51.7) |  |
| BOP (%) | | 21.1±21.2 | 37.1±25.5 | <0.01 |  | 17.7±18.4 | 29.0±25.0 | 50.2±20.4 | <0.01 |  | 8.5±8.4 | 30.0±19.6 | 60.5±20.2 | <0.01 |
| Max PD (mm) | | 2.5±0.6 | 3.0±1.0 | <0.01 |  | ― | ― | ― |  |  | 2.4±0.5 | 2.8±0.7 | 3.2±1.0 | <0.01 |
| Dental plaque | |  |  |  |  |  |  |  |  |  |  |  |  |  |
|  | No plaque | 69 (48.6) | 10 (16.9) | <0.01 |  | 46 (53.5) | 33 (33.0) | 0 (0.0) | <0.01 |  | ― | ― | ― |  |
|  | <1/3 | 59 (41.5) | 34 (57.6) |  |  | 34 (39.8) | 51 (51.0) | 8 (53.3) |  |  | ― | ― | ― |  |
|  | 1/3≤ | 14 (9.9) | 15 (25.4) |  |  | 6 (7.0) | 16 (16.0) | 7 (46.7) |  |  | ― | ― | ― |  |
| Tongue coating | |  |  |  |  |  |  |  |  |  |  |  |  |  |
|  | No coating | 42 (29.6) | 15 (25.4) | 0.27 |  | 21 (24.4) | 32 (32.0) | 4 (26.7) | 0.49 |  | 24 (30.4) | 27 (29.0) | 6 (20.7) | 0.38 |
|  | 0<, <1/3 | 48 (33.8) | 27 (45.8) |  |  | 33 (38.4) | 34 (34.0) | 8 (53.3) |  |  | 33 (41.8) | 33 (35.5) | 9 (31.0) |  |
|  | 1/3≤ | 52 (36.6) | 17 (28.8) |  |  | 32 (37.2) | 34 (34.0) | 3 (20.0) |  |  | 22 (27.8) | 33 (35.5) | 14 (48.3) |  |
| Dry mouth | |  |  |  |  |  |  |  |  |  |  |  |  |  |
|  | No | 55 (38.7) | 22 (37.3) | 0.85 |  | 27 (31.4) | 44 (44.0) | 6 (40.0) | 0.21 |  | 25 (31.6) | 39 (41.9) | 13 (44.8) | 0.28 |
|  | Yes | 87 (61.3) | 37 (62.7) |  |  | 59 (68.6) | 56 (56.0) | 9 (60.0) |  |  | 54 (68.4) | 54 (58.1) | 16 (55.2) |  |
| SBP (mmHg) | | 95.5±7.7 | 98.2±8.4 | 0.04 |  | 94.5±7.5 | 96.8±7.8 | 103.3±8.3 | <0.01 |  | 94.6±7.4 | 97.5±7.9 | 97.2±9.1 | 0.04 |
| DBP (mmHg) | | 46.7±7.0 | 48.5±7.6 | 0.13 |  | 47.2±7.0 | 47.0±7.5 | 49.7±6.2 | 0.40 |  | 47.2±7.6 | 47.8±7.2 | 45.7±6.1 | 0.38 |
| CAVI | | 4.7±0.6 | 4.6±0.6 | 0.25 |  | 4.7±0.6 | 4.6±0.6 | 4.6±0.5 | 0.14 |  | 4.7±0.7 | 4.7±0.5 | 4.5±0.5 | 0.43 |
| Passive smoking | |  |  |  |  |  |  |  |  |  |  |  |  |  |
|  | No | 116 (81.7) | 50 (84.7) | 0.60 |  | 70 (81.4) | 84 (84.0) | 12 (80.0) | 0.86 |  | 66 (83.5) | 77 (82.8) | 23 (79.3) | 0.87 |
|  | Yes | 26 (18.3) | 9 (15.3) |  |  | 16 (18.6) | 16 (16.0) | 3 (20.0) |  |  | 13 (16.5) | 16 (17.2) | 6 (20.7) |  |
| Maternal education | |  |  |  |  |  |  |  |  |  |  |  |  |  |
|  | Junior high school/high school | 26 (18.4) | 13 (22.0) | 0.01 |  | 22 (25.6) | 12 (12.0) | 5 (33.3) | 0.07 |  | 10 (12.7) | 21 (22.6) | 8 (27.6) | 0.10 |
|  | Specialized high school/vocational school/college | 50 (35.5) | 32 (54.2) |  |  | 29 (33.7) | 47 (47.0) | 6 (40.0) |  |  | 30 (38.0) | 38 (40.9) | 14 (48.3) |  |
|  | University/graduate school | 66 (46.5) | 14 (23.7) |  |  | 35 (40.7) | 41 (41.0) | 4 (26.7) |  |  | 39 (49.4) | 34 (36.6) | 7 (24.1) |  |
| Estimated daily salt intake (g) | | 6.5±1.1 | 6.3±1.4 | 0.20 |  | 6.3±1.2 | 6.7±1.2 | 6.4±1.3 | 0.06 |  | 6.3±1.2 | 6.6±1.2 | 6.5±1.2 | 0.30 |

B. Mothers

|  |  | D | |  |  | Periodontitis | |  |  |  | Dental plaque | | |  |
| --- | --- | --- | --- | --- | --- | --- | --- | --- | --- | --- | --- | --- | --- | --- |
|  |  | 0 | 1≤ |  |  | No | Yes |  |  |  | No plaque | 0, <1/3 | 1/3≤ |  |
| Variable | | n=157 | n=44 | *p* |  | n=124 | n=77 | *p* |  |  | n=100 | n=88 | n=13 | *p* |
| Age (years) | | 40.4±4.4 | 39.9±4.9 | 0.56 |  | 40.1±4.3 | 40.6±4.8 | 0.48 |  |  | 40.0±4.4 | 40.6±4.5 | 40.3±4.5 | 0.65 |
| BMI (kg/m^2^) | | 22.1±3.8 | 22.2±2.9 | 0.87 |  | 21.5±3.0 | 23.0±4.4 | 0.01 |  |  | 22.0±3.1 | 22.0±4.1 | 23.8±4.0 | 0.20 |
| DMF | |  |  |  |  |  |  |  |  |  |  |  |  |  |
|  | D=0 | ― | ― |  |  | 95 (76.6) | 62 (80.5) | 0.51 |  |  | 80 (80.0) | 68 (77.3) | 9 (69.2) | 0.66 |
|  | D≥1 | ― | ― |  |  | 29 (23.4) | 15 (19.5) |  |  |  | 20 (20.0) | 20 (22.7) | 4 (30.8) |  |
| BOP (%) | | 28.9±24.3 | 32.7±28.6 | 0.54 |  | 13.3±11.0 | 56.3±18.1 | <0.01 |  |  | 15.4±17.1 | 40.1±21.4 | 69.5±25.9 | <0.01 |
| Max PD (mm) | | 3.9±0.9 | 4.2±1.5 | 0.17 |  | 3.4±0.6 | 4.8±1.1 | <0.01 |  |  | 3.5±0.7 | 4.3±1.2 | 4.9±0.8 | <0.01 |
| Periodontitis | |  |  |  |  |  |  |  |  |  |  |  |  |  |
|  | No | 95 (60.5) | 29 (65.9) | 0.51 |  | ― | ― |  |  |  | 85 (85.0) | 38 (43.2) | 1 (7.7) | <0.01 |
|  | Yes | 62 (39.5) | 15 (34.1) |  |  | ― | ― |  |  |  | 15 (15.0) | 50 (56.8) | 12 (92.3) |  |
| Dental plaque | |  |  |  |  |  |  |  |  |  |  |  |  |  |
|  | No plaque | 80 (51.0) | 20 (45.5) | 0.66 |  | 85 (68.5) | 15 (19.5) | <0.01 |  |  | ― | ― | ― |  |
|  | <1/3 | 68 (43.3) | 20 (45.5) |  |  | 38 (30.6) | 50 (64.9) |  |  |  | ― | ― | ― |  |
|  | 1/3≤ | 9 (5.7) | 4 (9.1) |  |  | 1 (0.8) | 12 (15.6) |  |  |  | ― | ― | ― |  |
| Tongue coating | |  |  |  |  |  |  |  |  |  |  |  |  |  |
|  | No coating | 44 (28.0) | 10 (22.7) | 0.32 |  | 39 (31.5) | 15 (19.5) | 0.12 |  |  | 32 (32.0) | 21 (23.9) | 1 (7.7) | <0.01 |
|  | 0<, <1/3 | 54 (34.4) | 19 (43.2) |  |  | 46 (37.1) | 27 (35.1) |  |  |  | 34 (34.0) | 38 (43.2) | 1 (7.7) |  |
|  | 1/3≤, <2/3 | 39 (24.8) | 13 (29.5) |  |  | 29 (23.4) | 23 (29.9) |  |  |  | 25 (25.0) | 18 (20.5) | 9 (69.2) |  |
|  | 2/3≤ | 20 (12.7) | 2 (4.5) |  |  | 10 (8.1) | 12 (15.6) |  |  |  | 9 (9.0) | 11 (12.5) | 2 (15.4) |  |
| Dry mouth | |  |  |  |  |  |  |  |  |  |  |  |  |  |
|  | No | 57 (36.6) | 11 (25.0) | 0.16 |  | 44 (35.5) | 24 (31.2) | 0.53 |  |  | 34 (34.0) | 30 (34.1) | 4 (30.8) | 0.97 |
|  | Yes | 100 (63.7) | 33 (75.0) |  |  | 80 (64.5) | 53 (68.8) |  |  |  | 66 (66.0) | 58 (65.9) | 9 (69.2) |  |
| SBP (mmHg) | | 106.0±14.7 | 112.7±21.2 | 0.05 |  | 104.8±14.2 | 111.9±18.9 | 0.01 |  |  | 104.9±12.5 | 109.0±19.5 | 117.3±17.7 | 0.02 |
| DBP (mmHg) | | 68.4±10.1 | 72.5±12.4 | 0.05 |  | 67.2±9.7 | 72.6±11.5 | <0.01 |  |  | 68.4±9.7 | 69.1±11.4 | 77.2±11.0 | 0.02 |
| CAVI | | 6.7±0.6 | 6.7±0.8 | 0.70 |  | 6.6±0.7 | 6.8±0.6 | 0.08 |  |  | 6.5±0.7 | 6.8±0.6 | 6.8±0.5 | <0.01 |
| Smoking | |  |  |  |  |  |  |  |  |  |  |  |  |  |
|  | Non smoker | 121 (77.1) | 37 (84.1) | 0.01 |  | 98 (79.0) | 60 (77.9) | 0.31 |  |  | 74 (74.0) | 76 (86.4) | 8 (61.5) | 0.08 |
|  | Former smoker | 31 (19.7) | 2 (4.5) |  |  | 22 (17.7) | 11 (14.3) |  |  |  | 21 (21.0) | 9 (10.2) | 3 (23.1) |  |
|  | Smoker | 5 (3.2) | 5 (11.4) |  |  | 4 (3.2) | 6 (7.8) |  |  |  | 5 (5.0) | 3 (23.1) | 2 (15.4) |  |
| Drinking habits^a^ | |  |  |  |  |  |  |  |  |  |  |  |  |  |
|  | Non drinker | 89 (56.7) | 24 (54.5) | 0.80 |  | 68 (54.8) | 45 (58.4) | 0.62 |  |  | 59 (59.0) | 44 (50.0) | 10 (76.9) | 0.14 |
|  | Drinker | 68 (43.3) | 20 (45.5) |  |  | 56 (45.2) | 32 (41.6) |  |  |  | 41 (41.0) | 44 (50.0) | 3 (23.1) |  |
| Maternal education | |  |  |  |  |  |  |  |  |  |  |  |  |  |
|  | Junior high school/high school | 27 (17.2) | 13 (29.5) | 0.10 |  | 22 (17.7) | 18 (23.4) | 0.22 |  |  | 23 (23.0) | 15 (17.0) | 2 (15.4) | 0.67 |
|  | Specialized high school/ vocational school/college | 66 (42.0) | 12 (27.3) |  |  | 45 (36.3) | 33 (42.9) |  |  |  | 36 (36.0) | 35 (39.8) | 7 (53.8) |  |
|  | University/graduate school | 64 (40.8) | 19 (43.2) |  |  | 57 (46.0) | 26 (33.8) |  |  |  | 41 (41.0) | 38 (43.2) | 4 (30.8) |  |
| Estimated daily salt intake (g) | | 8.1±1.7 | 8.3±1.8 | 0.45 |  | 7.9±1.5 | 8.6±1.9 | <0.01 |  |  | 8.0±1.5 | 8.3±1.9 | 8.1±1.9 | 0.71 |

Continuous variables, mean±SD; categorical variables, n (%)

Student’s t-test, chi-squared test, Mann-Whitney U test, one-way analysis of variance or Kruskal-Wallis test were conducted according to the oral hygiene categories.

^a^Non-drinkers were defined as those who drank less than once per month

Supplemental Table 4. Odds ratios for high blood pressure in children defined using the simplified table

|  |  |  | **Complete case (n=201)** | | | | |  |  | **Multiply imputed (n=220)** | | | | |  |  | **Complete pairs (n=185) ^a^** | |
| --- | --- | --- | --- | --- | --- | --- | --- | --- | --- | --- | --- | --- | --- | --- | --- | --- | --- | --- |
|  |  | High BP | Model 1 (univariable) | |  | Model 2 ^b^ (multivariable) | |  | High BP | Model 1 (univariable) | |  | Model 2 ^b^ (multivariable) | |  | High BP | Model 3^c^ (multivariable) | |
| Variable | | (n) | OR | 95% CI |  | aOR | 95% CI |  | (n) | OR | 95% CI |  | aOR | 95% CI |  | (n) | aOR | 95% CI |
| d/D≥1 | | 10 | 1.73 | 0.73–4.11 |  | 1.43 | 0.58–3.56 |  | 11 | 1.68 | 0.74–3.85 |  | 1.46 | 0.62–3.46 |  | 10 | 1.24 | 0.46–3.33 |
| BOP≥10％ | | 19 | 1.81 | 0.69–4.76 |  | 1.52 | 0.56–4.09 |  | 21 | 1.99 | 0.77–5.17 |  | 1.82 | 0.69–4.78 |  | 18 | 1.33 | 0.47–3.77 |
| Max PD 3 mm | | 12 | 1.54 | 0.58–4.10 |  | 1.34 | 0.49–3.63 |  | 14 | 1.85 | 0.71–4.80 |  | 1.61 | 0.61–4.25 |  | 12 | 1.28 | 0.45–3.63 |
|  | 4 mm≤ | 6 | 7.52 | 2.07–27.34 |  | 5.18 | 1.33–20.23 |  | 6 | 6.21 | 1.79–21.61 |  | 4.94 | 1.35–18.09 |  | 5 | 3.68 | 0.87–15.66 |
| Dental plaque≥1/3 | | 6 | 2.10 | 0.76–5.81 |  | 1.06 | 0.71–1.58 |  | 6 | 1.76 | 0.65–4.75 |  | 1.75 | 0.63–4.86 |  | 6 | 1.83 | 0.58–5.79 |
| Tongue coating^d^ | | 15 | 0.55 | 0.23–1.30 |  | 0.61 | 0.25–1.49 |  | 17 | 0.61 | 0.26–1.42 |  | 0.70 | 0.29–1.70 |  | 15 | 0.58 | 0.22–1.52 |
| Dry mouth^e^ | | 16 | 1.12 | 0.47–2.68 |  | 1.11 | 0.45–2.71 |  | 17 | 1.12 | 0.47–2.68 |  | 0.99 | 0.42–2.32 |  | 16 | 1.33 | 0.51–3.47 |

^a^Complete child-mother pairs

^b^Adjusted for the propensity score calculated based on age (months), body weight, passive smoking status, and maternal educational background

^c^Adjusted for the propensity score calculated based on age (months), body weight, passive smoking status, maternal educational background, and propensity score of maternal oral hygiene

^d^Odds ratio for the presence of tongue coating was calculated with no coating as the reference

^e^Less than the cut-off value of 28.0 or not

aOR, adjusted OR; BOP, bleeding on probing; CI, confidence interval; OR, odds ratio; PD, pocket depth

Supplemental Table 5. Odds ratios of maternal oral hygiene parameters for high blood pressure in children defined using the simplified table

|  |  | **Complete pairs (n=185)** | | | | |
| --- | --- | --- | --- | --- | --- | --- |
|  | High BP | Model 1 (univariable) | |  | Model 2 ^a^ (multivariable) | |
| Variable | (n) | OR | 95% CI |  | aOR | 95% CI |
| D≥1 | 5 | 0.85 | 0.30–2.43 |  | 0.82 | 0.29–2.37 |
| Periodontitis | 11 | 1.50 | 0.63–3.57 |  | 1.25 | 0.50–3.08 |
| Dental plaque≥1/3 | 5 | 5.79 | 1.67–20.06 |  | 5.25 | 1.48–18.61 |
| Tongue coating | 23 | 10.36 | 1.36–78.87 |  | 10.59 | 1.38–81.07 |
| Dry mouth ^b^ | 18 | 1.56 | 0.58–4.15 |  | 1.47 | 0.55–3.94 |

^a^Adjusted for the propensity score calculated based on the maternal age, maternal BMI, and maternal estimated daily salt intake

^b^Less than the cut-off value of 28.0 or not

aOR, adjusted OR; CI, confidence interval; OR, odds ratios

Supplemental Table 6. Odds ratios of halitosis-causing substances for high blood pressure

| **Children** |  | **Complete case (n=201)** | | | | |  |  | **Multiply imputed (n=220)** | | | | |
| --- | --- | --- | --- | --- | --- | --- | --- | --- | --- | --- | --- | --- | --- |
|  | High BP | Model 1 (univariable) | |  | Model 2 ^a^ (multivariable) | |  | High BP | Model 1 (univariable) | |  | Model 2 ^a^ (multivariable) | |
| Variable | (n) | OR | 95% CI |  | aOR | 95% CI |  | (n) | OR | 95% CI |  | aOR | 95% CI |
| Hydrogen sulfide | 10 | 2.16 | 0.46–10.17 |  | 1.96 | 0.41–9.42 |  | 10 | 2.27 | 0.48–10.67 |  | 1.94 | 0.40–9.28 |
| Methyl mercaptan | 8 | 1.18 | 0.34–4.05 |  | 1.36 | 0.37–5.05 |  | 8 | 1.25 | 0.37–4.29 |  | 1.43 | 0.39–5.29 |
| Dimethyl sulfide | 8 | 0.61 | 0.17–2.11 |  | 0.46 | 0.13–1.70 |  | 8 | 0.63 | 0.18–2.19 |  | 0.51 | 0.14–1.86 |
| **Mothers** |  | **Complete case (n=201)** | | | | |  |  | **Multiply imputed (n=217)** | | | | |
|  | Hypertension | Model 1 (univariable) | |  | Model 2 ^b^ (multivariable) | |  | Hypertension | Model 1 (univariable) | |  | Model 2 ^b^ (multivariable) | |
| Variables | (n) | OR | 95% CI |  | aOR | 95% CI |  | (n) | OR | 95% CI |  | aOR | 95% CI |
| Hydrogen sulfide | 14 | 0.91 | 0.42–1.94 |  | 0.81 | 0.36–1.84 |  | 14 | 0.83 | 0.40–1.74 |  | 0.82 | 0.36–1.84 |
| Methyl mercaptan | 11 | 0.76 | 0.34–1.68 |  | 0.76 | 0.33–1.75 |  | 11 | 0.70 | 0.32–1.52 |  | 0.77 | 0.33–1.78 |
| Dimethyl sulfide | 20 | 0.62 | 0.28–1.38 |  | 0.63 | 0.27–1.47 |  | 20 | 0.58 | 0.28–1.21 |  | 0.68 | 0.30–1.55 |

The cut-off concentrations (ppb) for hydrogen sulfide, methyl mercaptan, and dimethyl sulfide were 112, 26, and 8, respectively

^a^Adjusted for propensity score calculated using age (months), body weight, passive smoking status, and maternal educational background

^b^Adjusted for propensity score calculated using age, BMI, and estimated daily salt intake

aOR, adjusted OR; CI, confidence interval; ORs, odds ratio

Supplemental Table 7. Odds ratios of halitosis-causing substances for high blood pressure in children as defined using the simplified table

|  |  | **Complete case (n=201)** | | | | |  |  | **Multiply imputed (n=220)** | | | | |
| --- | --- | --- | --- | --- | --- | --- | --- | --- | --- | --- | --- | --- | --- |
|  | High BP | Model 1 (univariable) | |  | Model 2 ^a^ (multivariable) | |  | High BP | Model 1 (univariable) | |  | Model 2^a^ (multivariable) | |
| Variable | (n) | OR | 95% CI |  | aOR | 95% CI |  | (n) | OR | 95% CI |  | aOR | 95% CI |
| Hydrogen sulfide | 22 | 3.42 | 0.98–11.91 |  | 3.91 | 1.07–14.29 |  | 24 | 3.97 | 1.15–13.68 |  | 4.03 | 1.15–14.17 |
| Methyl mercaptan | 17 | 1.27 | 0.52–3.12 |  | 1.45 | 0.57–3.71 |  | 19 | 1.54 | 0.64–3.70 |  | 1.73 | 0.69–4.31 |
| Dimethyl sulfide | 19 | 0.99 | 0.37–2.65 |  | 0.89 | 0.33–2.43 |  | 21 | 1.16 | 0.44–3.04 |  | 1.12 | 0.42–2.98 |

^a^Adjusted for propensity score calculated using age, body weight, passive smoking status, and maternal educational background

aOR, adjusted OR; CI, confidence interval; ORs, odds ratio

Supplemental Table 8. Odds ratios of oral hygiene indices for high blood pressure in children and their mothers adjusted for covariates individually.

A Children

|  |  | **Complete case (n=201)** | | |  | **Multiplicatively imputed dataset (n=220)** | | |  |  | **Complete pairs (n=185)^a^** | |
| --- | --- | --- | --- | --- | --- | --- | --- | --- | --- | --- | --- | --- |
|  |  | High BP | Model 2^b^ (multivariable) | |  | High BP | Model 2^b^ (multivariable) | |  | High BP | Model 3^c^ (multivariable) | |
| Variable |  | (n) | aOR | 95% CI |  | (n) | aOR | 95% CI |  | (n) | aOR | 95% CI |
| d/D≥1 | | 7 | 3.17 | 0.89 – 11.24 |  | 7 | 3.06 | 0.86 – 10.86 |  | 7 | 2.57 | 0.67 – 9.80 |
| BOP≥10% | | 9 | 1.29 | 0.32 – 5.27 |  | 9 | 1.37 | 0.34 – 5.45 |  | 9 | 1.07 | 0.25 – 4.63 |
| Max PD^d^ | 3 mm | 5 | 1.25 | 0.28 – 5.65 |  | 5 | 1.23 | 0.27 – 5.55 |  | 5 | 1.07 | 0.23 – 5.05 |
|  | 4 mm≤ | 4 | 8.20 | 1.37 – 49.14 |  | 4 | 8.23 | 1.42 – 47.71 |  | 4 | 8.13 | 1.34 – 49.22 |
| Dental plaque≥1/3 | | 3 | 2.09 | 0.49 – 9.00 |  | 3 | 2.2 | 0.52 – 9.41 |  | 3 | 1.55 | 0.31 – 7.89 |
| Tongue coating^e^ | | 7 | 0.61 | 0.18 – 2.10 |  | 7 | 0.60 | 0.17 – 2.06 |  | 7 | 0.49 | 0.13 – 1.84 |
| Dry mouth^f^ | | 8 | 1.29 | 0.36 – 4.57 |  | 8 | 1.21 | 0.34 – 4.31 |  | 8 | 1.21 | 0.33 – 4.49 |

B. Mothers

|  |  | **Complete case (n=201)** | | |  | **Multiplicatively imputed dataset (n=217)** | | |
| --- | --- | --- | --- | --- | --- | --- | --- | --- |
|  |  | Hypertension | Model 2^g^ (multivariable) | |  | Hypertension | Model 2^g^ (multivariable) | |
| Variables |  | (n) | aOR | 95% CI |  | (n) | aOR | 95% CI |
| D≥1 | | 11 | 2.21 | 0.94 – 5.18 |  | 13 | 2.69 | 1.13 – 6.41 |
| Periodontitis | | 18 | 1.82 | 0.81 – 4.09 |  | 21 | 2.08 | 0.91 – 4.75 |
| Dental plaque≥1/3 | | 6 | 4.73 | 1.40 – 15.99 |  | 7 | 4.76 | 1.36 – 16.64 |
| Tongue coating^e^ | | 27 | 2.21 | 0.79 – 6.21 |  | 30 | 2.12 | 0.74 – 6.11 |
| Dry mouth^f^ | | 26 | 2.21 | 0.79– 6.21 |  | 28 | 2.16 | 0.81 – 5.78 |

^a^Complete child-mother pairs

^b^Adjusted for age (months), body weight, passive smoking status, and maternal educational background

^c^Adjusted for age (months), body weight, passive smoking status, maternal educational background, and propensity score of maternal oral hygiene

^d^PD=2 mm was used as a reference (no participant had max PD = 1)

^e^No coating was used as the reference

^f^Less than the cut-off value of 28.0 or not

^g^Adjusted for age and BMI, and estimated daily salt intake

Supplemental Table 9. Odds ratios of maternal oral hygiene parameters associated with high blood pressure in children adjusted for covariates individually

|  |  | **Complete pairs (n=185)** | |
| --- | --- | --- | --- |
|  | High BP | Model 2^a^ (multivariable) | |
| Variables | (n) | aOR | 95% CI |
| D≥1 | 2 | NA^b^ | ― |
| Periodontitis | 5 | 1.04 | 0.30–3.63 |
| Dental plaque≥1/3 | 3 | 5.59 | 1.23–25.44 |
| Tongue coating | 12 | NA^b^ | ― |
| Dry mouth^c^ | 9 | 1.53 | 0.39–5.92 |

^a^Adjusted for maternal age, BMI, and estimated daily salt intake

^b^Not applicable as there were two or no pairs of a mother with D≥1 or no tongue coating and a child with high blood pressure, respectively.

^c^Less than the cut-off value of 28.0 or not

Supplemental Table 10. Estimated marginal means of the CAVI according to oral hygiene indices in children and mothers adjusted for covariates individually

|  | Variable |  | n (%) | Estimated marginal mean ± SE | F statistic | *p* |
| --- | --- | --- | --- | --- | --- | --- |
| Children | Max PD | 2 mm | 86 (42.8) | 4.7±0.1 | 1.85 | 0.16 |
|  |  | 3 mm | 100 (49.8) | 4.6±0.1 |  |  |
|  |  | 4 mm≤ | 15 (7.5) | 4.8±0.2 |  |  |
|  |  |  |  |  |  |  |
|  | d/D | 0 | 142 (70.6) | 4.7±0.1 | 0.52 | 0.47 |
|  |  | 1≤ | 59 (29.4) | 4.6±0.1 |  |  |
|  |  |  |  |  |  |  |
|  | Dental plaque | No plaque | 79 (39.3) | 4.7±0.1 | 0.67 | 0.51 |
|  |  | 0<, <1/3 | 93 (46.3) | 4.7±0.1 |  |  |
|  |  | 1/3≤ | 29 (14.4) | 4.5±0.1 |  |  |
| Mothers | Periodontitis | No | 124 (61.7) | 6.6±0.1 | 4.35 | 0.04 |
|  |  | Yes | 77 (38.3) | 6.8±0.1 |  |  |
|  |  |  |  |  |  |  |
|  | D | 0 | 157 (78.1) | 6.7±0.1 | 0.06 | 0.8 |
|  |  | 1≤ | 44 (21.9) | 6.7±0.1 |  |  |
|  |  |  |  |  |  |  |
|  | Dental plaque | No plaque | 100 (49.8) | 6.5±0.1 | 5.51 | <0.01 |
|  |  | 0<, <1/3 | 88 (43.8) | 6.8±0.1* |  |  |
|  |  | 1/3≤ | 13 (6.5) | 6.9±0.2 |  |  |

ANCOVA was used to estimate marginal CAVI means among the exposure categories after adjusting for covariates.

Children were adjusted for age (month), sex, BMI, and estimated daily salt intake.

Mothers were adjusted for age, BMI, estimated daily salt intake, drinking habit, and smoking status.

*p<0.01 vs no plaque group


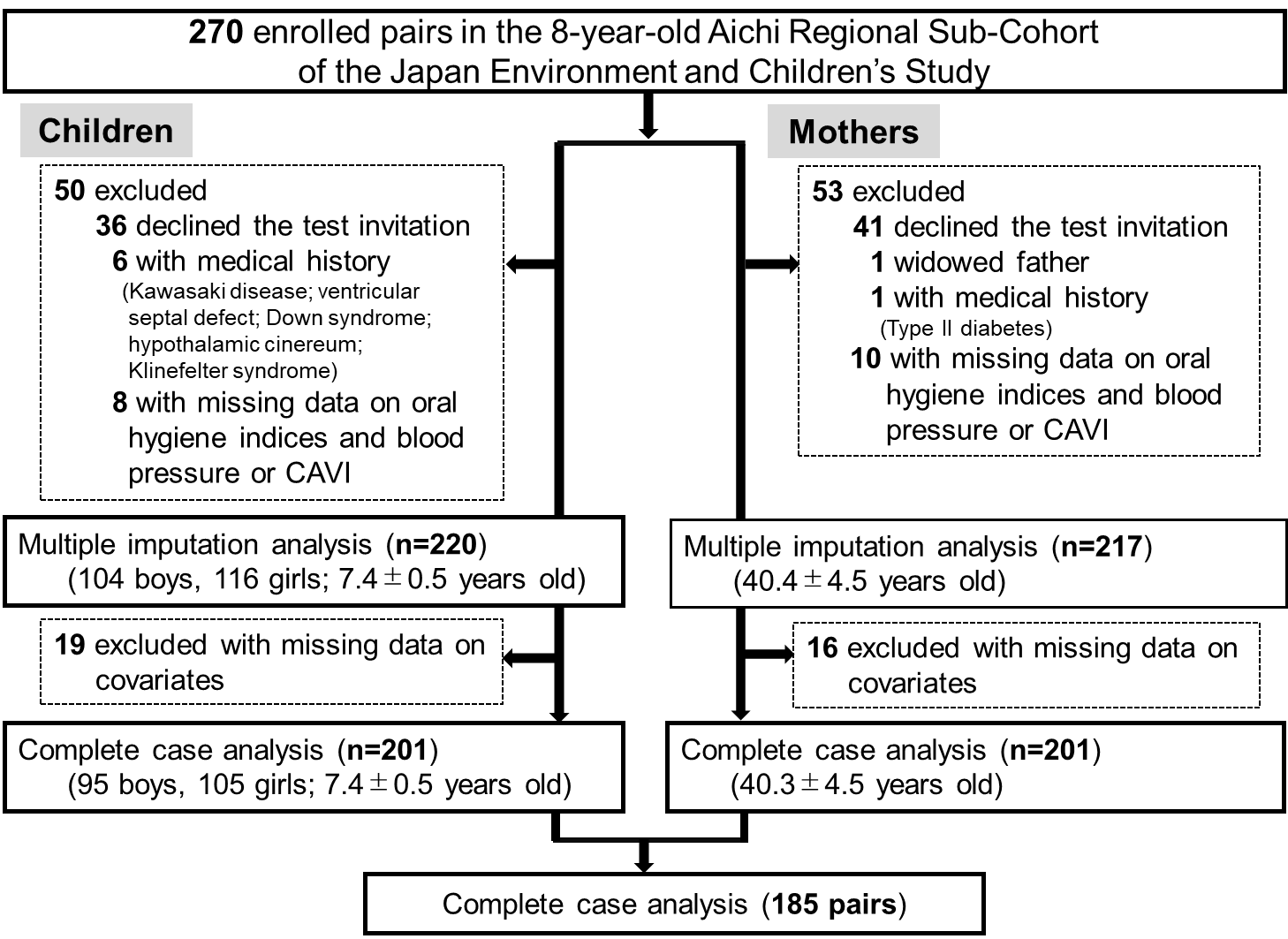


Supplemental Fig. 1. Study participant selection flowchart


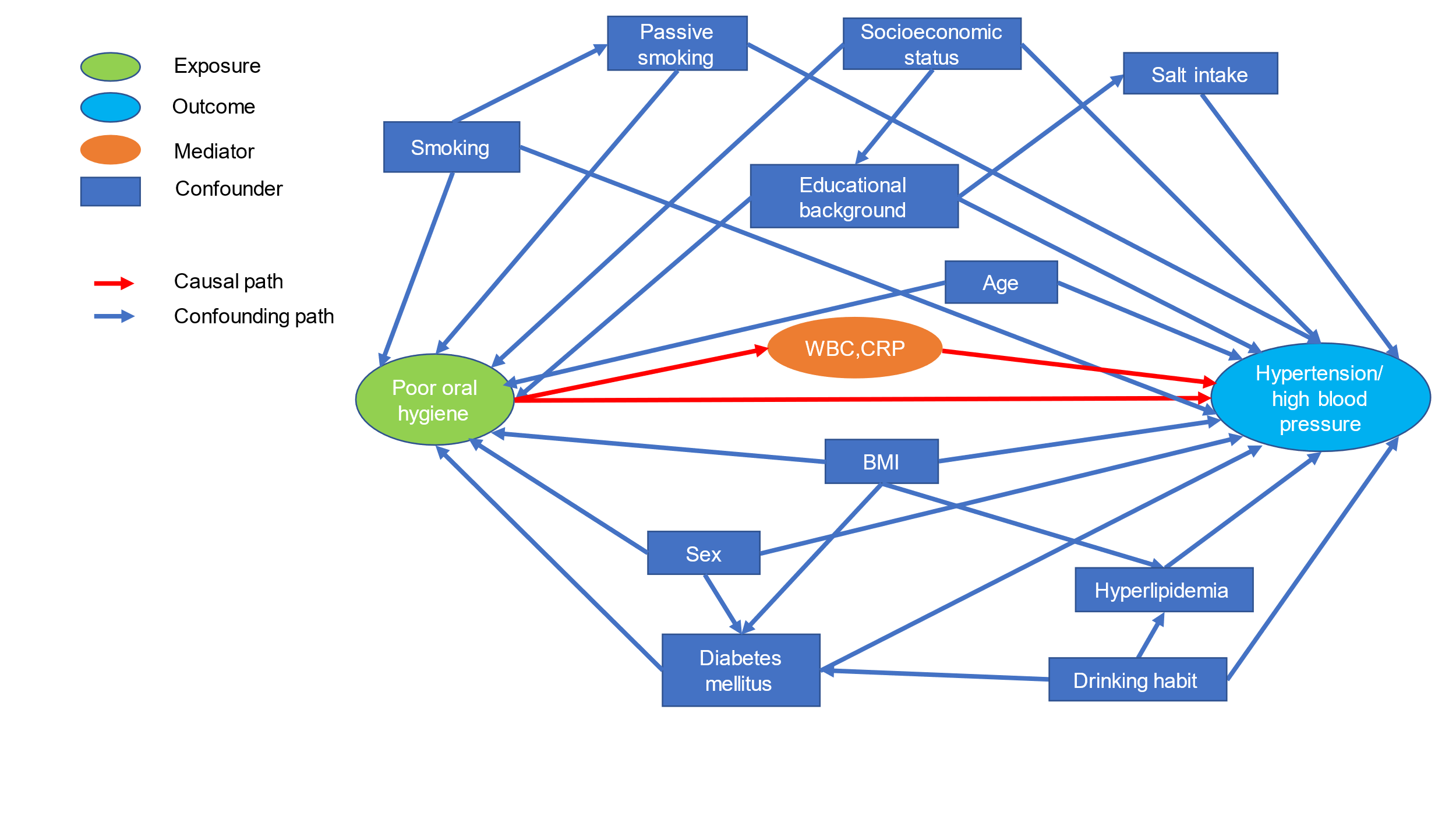


Supplemental Fig. 2. Directed acyclic graph used for the analyses

WBC, white blood cell; CRP, C-reactive protein; BMI, body mass index


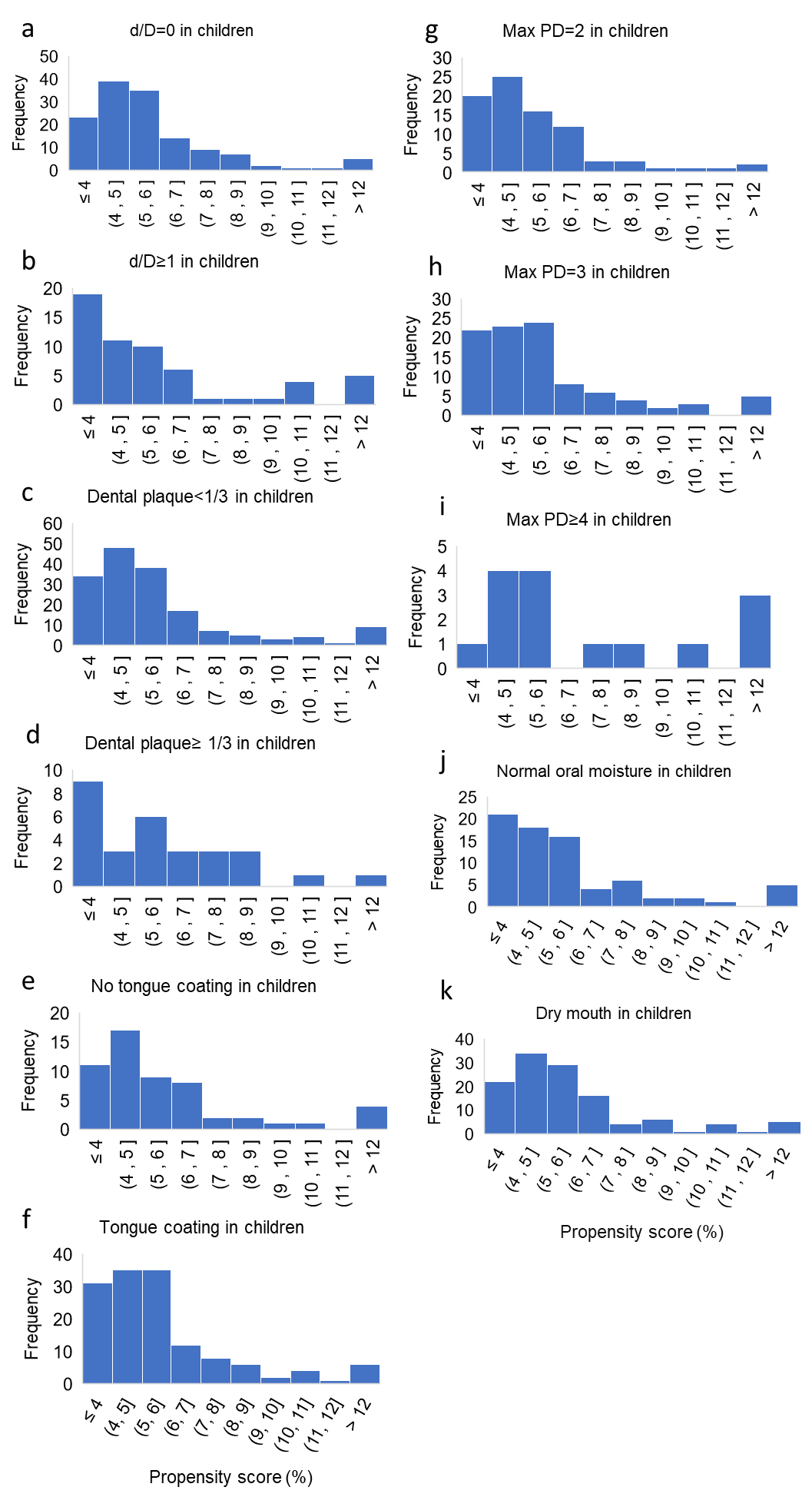


Supplemental Fig. 3. Distribution of propensity scores per group in children’s oral hygiene indices.

dental cavity (a, b), dental plaque (c, d, e), tongue coating (e, f), max pocket depth (g, h, i), dry mouth (j, k)


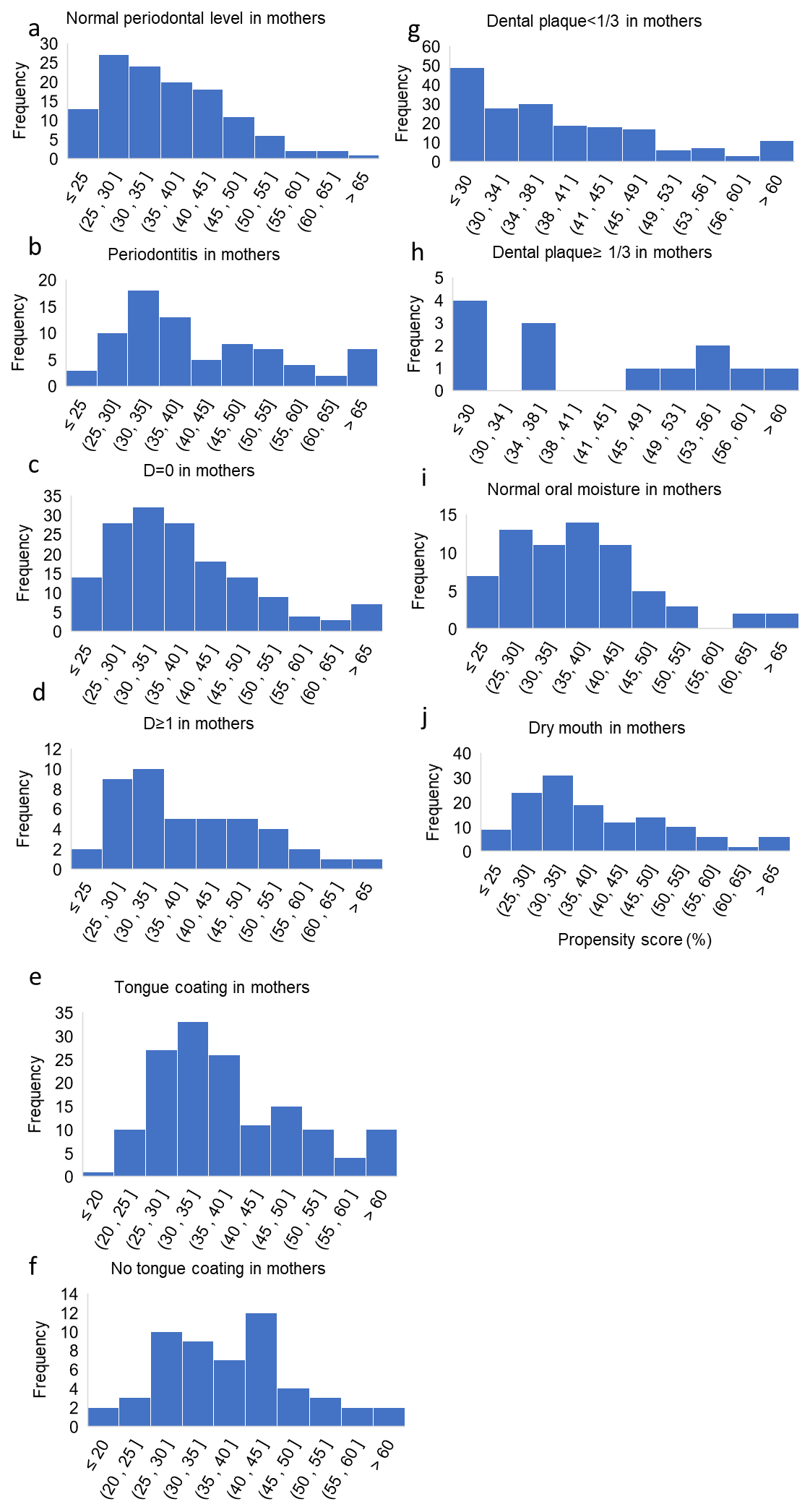


Supplemental Fig. 4. Distribution of propensity scores per group in mother’s oral hygiene indices.

periodontitis (a, b), dental cavity (c, d), tongue coating (e, f), dental plaque (g, h), dry mouth (i, j)
